# Strategy for universal access to health and universal health coverage and the contribution of the International Nursing Networks^1^

**DOI:** 10.1590/0104-1169.0000.2502

**Published:** 2014

**Authors:** Silvia Helena De Bortoli Cassiani

**Affiliations:** Regional Advisor for Nursing and Health Technicians, Pan American Health Organization/World Health Organization, Washington, DC, United States

Last October 2014, the 53rd Directing Council of the Pan American Health Organization, including the ministers of health or their representatives, of all countries in the Americas, approved Resolution CP53.14 about the "Strategy for universal access to health and universal health coverage"^(^ 1 ^)^.

Health coverage is defined as "the capacity of the health system to serve the needs of the population, including the availability of infrastructure, human resources, health technologies (including medicines) and financing. Universal access is defined as the absence of geographical, economic, sociocultural, organizational or gender barriers"^(^ 1 ^)^. Universal access is achieved through the progressive elimination of the barriers that impede all people from using the integral health services, equitably established at the national level.

The universal access to health and the universal health coverage are necessary to improve the health results and other fundamental objectives of the health systems, and are based on all people's right to enjoy the maximum level of health, equality and solidarity. The universal health coverage strategy is being used to bring all program interests in health under an inclusive umbrella and explain its relation with the increased healthy life expectancy, according to the most recent discussions on the millennium development goals after 2015^(^ 2 ^)^.

The human resources in health are one of the central pillars for the Universal Access to Health and Universal Health Coverage. Nevertheless, profound disequilibria and gaps remain in the availability, distribution, composition, competency and productivity of the human resources in health, mainly in primary care. Eleven countries in the region face an absolute shortage of health professionals (less than 25 physicians, nurses and certified midwives per 10,000 inhabitants). To expand the effective and equitable health coverage, many countries in Latin America need to improve the training and distribution of human resources in health^(^ 3 ^)^.

Nursing plays a fundamental role for the countries to achieve the target of Universal Health Coverage and Universal Access to health services. Nurses and nursing personnel can act in health services at all care levels. The nurses' education should prepare them to use and apply scientific knowledge, for the critical and reflexive analysis of their professional practice and context, and for the use of technical, scientific and interpersonal relationship skills in human care.

Challenges remain internal and external to the profession. These challenges, some of which have historical backgrounds, are cultural, related to gender and knowledge and the tireless struggle for a professional space that is not always acknowledged, attributed and valued inside the health system. The nurses and nursing personnel in most countries are insufficient and badly distributed, in combination with a lack of motivation and a performance that can improve in terms of quality and patient safety. The nursing care is not always delivered by professionals with further technical and scientific background, so that the population does not distinguish the nurse as the professional with a university background. In addition, there exists tension for the workspace among the groups inside the profession.

Nevertheless, one fortress should be acknowledged, which is the ability, desire and strength of the profession to work jointly to achieve a target. The creation and success of communities of practice in nursing in Latin America, better known as the International Nursing Networks, coordinated by the Pan American Health Organization/World Health Organization, are an example of this strength of nurses.

The desire to contribute, to make themselves known, to exchange knowledge and activities, to associate and grow collectively, has produced an intense social and political movement among nurses, mainly those coming from the universities, in the countries of the Latin-America, through the national and international nursing networks.

PAHO/WHO knew how to recognize, support and stimulate at the right time the work that nurses' organization in networks has been developing and which has constituted more than 25 international networks so far, joining more than 3000 nurses.

Because of this background and work, the international nursing networks can and should be acknowledged as a potential and as one of the contributions of nursing and nurses to achieve universal health coverage and universal health access for all people.
